# Identification of miRNAs Involved in Foetal Growth Restriction Due to Maternal Smoking during Pregnancy

**DOI:** 10.3390/jcm11195808

**Published:** 2022-09-30

**Authors:** Eva Barrio, Alba Quirós, Diego Lerma-Puertas, José I. Labarta, Ana Gascón-Catalán

**Affiliations:** 1 Facultad de Medicina, Universidad de Zaragoza, 50009 Zaragoza, Spain; 2 Servicio de Obstetricia y Ginecología, Hospital Clínico Universitario Lozano Blesa, 50009 Zaragoza, Spain; 3 Unidad de Endocrinología, Servicio de Pediatría, Hospital Universitario Miguel Servet, 50009 Zaragoza, Spain; 4 Facultad de Ciencias de la Salud, Universidad de Zaragoza, 50009 Zaragoza, Spain

**Keywords:** epigenetic modifications, miRNA, tobacco, intrauterine growth restriction

## Abstract

Introduction: Smoking during pregnancy is associated with reduced foetal growth, amongst other effects. Epigenetic modification in the foetus and placenta during embryonic development as a result of changes in the function of miRNAs is one of the pathophysiological mechanisms responsible for this. This dysregulation may be due to environmental changes or toxins such as tobacco. Objective: To study the impact of smoking during pregnancy and its role in intrauterine growth restriction via hypermethylated miRNAs. Materials and methods: The differences in methylation patterns for miRNAs in umbilical cord blood from low-birth-weight newborns of smoking mothers were compared with those from normal-weight newborns using MedIP-seq (StarArray). Results: Seven hypermethylated miRNAs were identified in the epigenetic study of cord blood from low-birth-weight newborns of smoking mothers in our sample. The miRNAs found to be hypermethylated were: MIR7-1, MIR3918, MIR1244-1, MIR4721, MIR25, MIR93, MIR3656. Conclusion: Intrauterine exposure to tobacco induces hypermethylation-mediated miRNA silencing in low-birth-weight newborns by modifying the expression of factors involved in vascular development, growth, and adaptation to hypoxia.

## 1. Introduction

Epigenetics refers to the modification of gene expression without altering the DNA sequence [1,2,3,4]. DNA methylation is one of the main mechanisms involved in regulating gene expression. Under normal conditions, methylation patterns are specific for each tissue in the body and, therefore, for each species, and can be inherited. During embryonic development (from the eighth cell division onwards), cells undergo new methylation processes, which may also appear in somatic cells of adults. An inversely proportional relationship can be established between DNA methylation and gene expression [1] and may be subject to changes induced by the environment or external toxic factors, such as tobacco, malnutrition or contamination [2,4,5].

Intrauterine growth restriction (IUGR) is a heterogeneous entity that encompasses numerous aetiologies and requires a factor inhibiting intrauterine foetal growth. Its incidence varies between populations, and it is estimated that around 30 million children are born annually worldwide with a low weight for their gestational age. Maternal malnutrition is the leading cause of IUGR worldwide, followed by the intake of toxins, especially tobacco, both actively and passively [6]. A study conducted by our group revealed that the most significant idiopathic aetiological factors related to IUGR are tobacco, maternal stress, total number of months worked during pregnancy, hours worked per day and time standing [7].

Tobacco is a negative factor for foetal growth. Smoking affects length, weight and head circumference and its effects can be detected by week 30 of pregnancy, with a weight reduction of approximately 250 g having been reported. However, the literature on the link between maternal smoking and restricted foetal growth is not completely consistent, as a reduction in foetal growth has been reported in some, but not all, studies, and others have documented direct tissue damage and impaired placental circulation [8].

Epidemiological studies in humans and experimental animals have shown that a suboptimal intrauterine and breastfeeding environment affects growth and predisposes the newborn to other diseases in adult life. Maternal smoking during pregnancy may modify the methylation pattern of foetal DNA in genes involved in foetal growth and development [9] and may also induce epigenetic modifications and alter gene expression during pregnancy, thereby affecting prenatal growth, foetal and neonatal phenotype and susceptibility to developing diseases as adults such as diabetes mellitus, obesity, dyslipidaemia, asthma, behavioural disorders and cardiovascular disease, amongst others. A positive association between maternal smoking and obesity or overweight in offspring has been demonstrated, whereas the relationship with type 2 diabetes remains uncertain [10].

Epigenomic wide association studies (EWAS) have shown the association between tobacco and DNA methylation in cord blood and newborns, with some of these alterations being reversible postnatally. In addition, recent studies have shown that some of these modifications to the DNA methylation pattern may persist postnatally until adolescence [11]. The identification of epigenetic changes associated with tobacco exposure could be used in the future to identify the population susceptible to developing chronic diseases as adults. As such, study of the methylation in these regions may be an indicator of prenatal harm. The possible reversibility of these epigenetic modifications opens up a window of opportunity to modulate and change the evolution of prenatally “programmed” chronic diseases.

Exposure to tobacco smoke during pregnancy may be associated with an increase in miRNA genes methylation and therefore altered miRNA expression in newborns [12].

miRNAs regulate gene expression post-transcriptionally by inducing gene silencing. They bind to messenger RNAs due to their complementarity, thereby preventing their translation [4]. They also act as internal regulators for a large number of genes, including the retrotransposons required for the stability of DNA or regulation of processes such as cell growth and differentiation [13].

Between 1100 and 1800 miRNA-coding genes are currently known in humans and mice, although they are also found in numerous other species. These genes are involved in several physiological (control of the cell cycle and division and tissue growth and differentiation) and pathological processes (cancer and neurological diseases) [14].

The role of miRNAs in growth has been studied in various animal models. Analysis of altered miRNAs in human growth impairment suggests that miRNAs contribute to growth physiology by regulating the hypothalamic-pituitary-IGF axis, growth hormones (GH), insulin-like growth factors (IGF) and their related receptors [15].

miRNAs can cross the placental barrier and reach foetal blood. Their regulation occurs in maternal, foetal and placental tissue. Four miRNAs that are specifically expressed in the placenta—miRNA-141, miRNA-149, miRNA-229-5p and miRNA-135b—and can be detected in both maternal and foetal blood [16].

Furthermore, one of the factors known to cause placental dysfunction leading to the onset of IUGR is exposure to tobacco smoke, which is associated with an increase in neonatal mortality and morbidity and is also related to an increased risk of developing adult diseases in exposed children [17]. The methylation processes resulting from nicotine exposure can trigger a decrease in the expression of genes that are key to correct development of the placenta and uteroplacental arteries, as well as genes that are essential for adequate foetal growth. Nicotine also acts directly on placental acetylcholine receptors, causing vasoconstriction and increasing uterine vascular resistance, thereby resulting in decreased foetal blood flow [18].

Given the relationship between tobacco use during pregnancy, the epigenetic modifications caused by this exposure and IUGR, the objectives of this study were:

1. To compare the difference in miRNA methylation in low-birth-weight newborns of smoking mothers with those in normal-weight newborns of non-smoking mothers;
2. To establish a relationship between tobacco, altered miRNA expression patterns and IUGR.

## 2. Materials and Methods

This is a transversal study conducted in newborns at the Hospital Clínico Universitario Lozano Blesa (Zaragoza, Spain). Sample collection commenced on 1 October 2018 and was completed on 30 December 2019. The sample comprised ten umbilical cord blood from newborns of smoking and non-smoking mothers.

### 2.1. Inclusion Criteria

1. Low-birth-weight newborns [19,20] exposed to tobacco smoke: children of mothers who smoked more than 10 cigarettes per day during the first trimester of pregnancy;
2. Normal-weight newborns not exposed to tobacco smoke: children of mothers who did not smoke during pregnancy.

### 2.2. Exclusion Criteria

1. Diseases in the mother that could cause IUGR;
2. Uterine infections;
3. Foetal malformations;
4. Chromosome abnormalities;
5. Premature birth;
6. Not signing informed consent.

### 2.3. Study Variables

1. Clinical variables for the mother and newborn (Table 1);
2. Study of differentially methylated miRNAs in umbilical cord blood

### 2.4. Procedure and Methodology

Patients who complied with the inclusion criteria were selected consecutively in the obstetrics clinic until obtaining the total sample size. After receiving explanations about the study, they were asked to sign the informed consent. The study was approved by the Ethical and Research Clinical Committee of the Health Research Institute of Aragon.

A cord blood sample was obtained during delivery and transferred to the Genetics laboratory at the School of Medicine of the University of Zaragoza for subsequent DNA extraction.

The genes to be studied had been selected previously by way of a MeDIP-seq (Methylated DNA immunoprecipitation sequencing) analysis.

For this analysis, a DNA sample was taken and cleaved into fragments of between 200 and 800 base pairs. These were then ligated to Illumina adapters and subsequently immunoprecipitated using the anti-5-methylcytosine antibody. The enriched DNA was amplified by PCR and purified. A subsequent quality control (QC) was performed.

The short fragments from the sequences generated using HiSeq4000 were then analysed from the 5′ to the 3′ end and aligned with the previously selected reference genome. A mapping was then performed thus obtaining the different hypermethylated sequences, the associated long non-coding RNA (ncRNA) fragments, the messenger RNA (mRNA) fragments and the small non-coding RNA (smallncRNA) fragments. The latter are part of the RNA silencing complex, which comprises the siRNA, piRNA and miRNA, with the latter being the subject of our study and of the analysis of results.

The percentage of cytosine methylation in the miRNA promoter sequences was quantified and the existence of significant differences between smoking habit and IUGR was analysed.

The selected methylated miRNA gene were analysed using the mirBASE and miRDB databases to determine in which regions of the genome they may act (TARGET) and induce gene silencing.

The mirBASE database is a platform that allows for searching published miRNA sequences and data annotation. Each entry contains the information for the hairpin precursor from which transcription of the different mature miRNAs is obtained, locating and classifying them on the basis of the region of the genome in which they act to induce post-transcriptional silencing. This database was created and is administered by the Griffiths-Jones laboratory in the Faculty of Biology, Medicine and Health at the University of Manchester.

After this initial search, another was conducted in the miRDB database. On this platform, each of the targets described according to the base complementarity of the different miRNAs studied was analysed, thereby predicting the functions of each of them.

## 3. Results

Seven hypermethylated miRNAs were identified in the genetic study of cord blood from low-birth-weight newborns of smoking mothers in our sample, namely MIR7-1, MIR3918, MIR1244-1, MIR4721, MIR25, MIR93, MIR3656. These are distributed on chromosomes 2, 6, 7, 9, 11 and 16.

After searching for possible targets for the miRNAs described in Table 2 and Table 3 using the mirRDB database, the most relevant in terms of foetal growth were selected. The possible targets for each of the miRNAs described can be found in Table 4, Table 5, Table 6, Table 7, Table 8, Table 9, Table 10 and Table 11. No targets were found for the sequence of miRNA3656.

Some of these miRNAs (MIR7-1, MIR25, MIR93, MIR3656) are primarily involved in diseases related to neurodegenerative alterations, breast tumours, stomach tumours and metabolic alterations.

With regard to foetal development, we have found targets for these miRNAs related to:

1. Placental angiogenesis, as Vascular Endothelial Growth Factor A (VEGFA) and Angiogenic Factor 1 (FA1);
2. Foetal and placental growth: insulin-like growth factor 2 mRNA binding protein 2 (IGF2BP2) and Placental Growth Factor (PLGF);
3. Compensatory mechanisms to foetal hypoxia: Hypoxia Inducible Factor 3 Subunit Alpha (HIF3A).

## 4. Discussion

Our study presents new findings concerning the relationship between miRNA regulation by methylation during pregnancy and foetal growth. We identified hypermethylation in seven miRNAs in cord blood from low-birth-weight newborns of smoking mothers with statistically significant differences with respect to normal-weight newborns of non-smoking mothers. These miRNAs are involved in foetal development by regulating processes such as placental angiogenesis, foetoplacental growth and foetal oxygenation mechanisms.

Exposure to tobacco during pregnancy affects the normal miRNA methylation patterns in foetuses, thereby inducing a decrease in gene expression in the regions affected, which in turn leads to deregulation of the synthesis of the resulting proteins [21,22].

miRNAs are essential regulatory elements in embryonic development. Alteration of the expression of the miRNAs found in our study (MIR7-1, MIR3918, MIR1244-1, MIR4721, MIR25, MIR93, MIR3656) may be associated with the decreased expression of proteins and key factors for correct placental development.

Firstly, we observed methylation-mediated silencing of miRNA-7-1 and miRNA-93 with targets in pro-angiogenic factors related to foetal and placental growth (VEGFA and PLGF). This should result in overexpression of these growth factors, which would in turn lead to normal foetal growth. However, we have observed this alteration of expression in foetuses with IUGR, which could be explained by an alteration of angiogenesis caused by the overexpression and related to the endothelial vascular dysfunction found in IUGR. Our findings are in agreement with those of Luo J et al., who found a statistically significant decrease in the expression of various families of miRNAs, especially miRNA-150, which exhibits pro-angiogenic activity in venous cord blood in foetuses with IUGR compared with the control group, in which the expression was normal [23]. A more recent study has also demonstrated the relationship between the expression of miRNA-206, the pathophysiology of IUGR and the vascular endothelial growth factor (VEGFA) in maternal blood [24].

Another possible target, in this case for the 3′ end of miRNA-25, found with regards to vascular dysfunction is angiogenic factor 1, which is related to correct blood vessel development [25].

Secondly, miRNA-3918 targets placental growth factor (PLGF) and human chorionic somatomammotropin hormone (CSHL1). These proteins are essential for correct development of the placenta and adequate evolution of pregnancy. A decrease in PLGF is related to the risk of suffering pre-eclampsia, the pathophysiology of which is closely related to that of IUGR. Indeed, PLGF is currently used clinically for the early diagnosis of pre-eclampsia and IUGR [26,27,28].

Surprisingly, we found hypermethylated miRNA-3918 in foetuses with restricted growth, which means that PLGF expression should be increased, a finding that contradicts what would be expected for a foetus with IUGR as, in such cases, PLGF concentration should be reduced. However, previous studies have reported similar findings, with an increase in PLGF in IUGR, meaning that this factor may behave in different ways depending on the degree of placental oxygenation and the interaction with other pro-angiogenic factors such as VEGFA [29,30]. This situation is similar to our findings for angiogenic factors.

Thirdly, the 5′ end of miRNA-93 and miRNA-3918 target insulin-like growth factor-binding protein 1 (IGF2BP-1) and 2 (IGF2BP-2), respectively, which are essential for correct foetal growth and have high expression in most foetal organs during embryogenesis. The proteins IGF2BP1 and IGF2BP2 interact with 5’-UTR of IGF2 and protect it from miRNA-mediated silencing [31].

IUGR is a consequence of placental vascular dysfunction, and several other factors are involved in its pathophysiology. The IGF system is one of the most important endocrine and paracrine growth factor system that regulate foetal and placental growth and IGF-2 plays a major role in preservation of placental growth and function. IGFBP-2 concentration in placental lysates resulted to be an important variable in determining foetal growth [32].

Altered expression of IGFs and IGF binding proteins during intrauterine growth retardation has been shown in experimental animal models with elevated plasma IGFBP-2 and IGFBP-4 and reduce IGFBP-3 [33]. IGFBP-2 is the principal IGFBP in foetal plasma and after uterine artery ligation a strong increase in the concentration of IGFBP-2 has been observed and this mimics the rise in IGFBP-1 and IGFBP-2 levels seen in human IUGR [34]. IGF-2 cord serum concentration has a positive effect on both birth length and weight, whereas IGFBP-2 had a significant negative effect [35]. The proportional increase in IGFBP-2 in the circulation of foetal growth restricted foetuses without changes in IGFs indicates that there might be a causal relationship between the increase in IGFBP-2 and foetal growth. The increase observed in IGFBP-2 in growth-restricted foetuses would be a compensatory mechanism for a greater IGF tissue bioavailability as IGFBP-2, in contrast to IGFBP-3, does not form a long-lived ternary complex with the IGFs [36] and our observation with higher expression of IGFBP-2 gene due to hypermethylation of miRNA-3918 is consistent with this hypothesis.

It has been shown that in the female sex-selective increase in IGF-2 expression in the hypoxic guinea pig placenta of growth-restricted foetuses as a mechanism of adaptation [37].

The reduced expression of IGF-2 in children with IUGR and its relationship to the altered expression of miRNAs have been reported in previous studies, as has its involvement in placental dysfunction [38].

Finally, hypoxia-inducible factor-3 is a protein coded by the HIF3 gene, which is a possible target for miRNA-4721. HIF3 plays a key role in the adaptive tissue response to situations of chronic hypoxia that lead to alterations in tissues and organs. Hypoxia-inducible factors (HIF1, 2 and 3) regulate oxygen homeostasis by controlling angiogenesis during periods of oxygen deficit. When HIF-1 levels decrease, HIF-2 and HIF-3 increase. This switch from HIF-1 to HIF-2 and HIF-3 signalling is required in order to adapt the endothelium to prolonged hypoxia. Recently, the miRNAs that endogenously regulate gene expression via the RNA interference (RNAi) pathway have been shown to play critical roles in the hypoxia response pathways [39].

miRNA-4721 methylation and the increased expression of HIF-3 may be an adaptive response to chronic oxygen deficit that occurs in IUGR as a result of altered tissue perfusion.

Our findings suggest a new perspective with regards to the role played by miRNAs in the regulation of angiogenic and placental growth factors and IUGR.

The results of our study agree with the foetal programming hypothesis proposed by Barker, who posits that foetuses with IUGR compensate for the hostile intrauterine environment by modifying their metabolism, which subsequently remains during postnatal development, thus leading to chronic diseases in adult life [40,41]. Consequently, these miRNAs may prove useful as biomarkers for the diagnosis and follow-up of these diseases. In light of our findings, we propose a new mechanism concerning the epigenetic regulation mechanisms of miRNAs and the foetoplacental angiogenic and growth factors essential for correct foetal development.

The main limitation with regards to the study of miRNAs lies in the lack of functional studies relating a specific miRNA to an associated pathology. Our study proposes a new explanation for the development of IUGR as well as a new line of research regarding the role of miRNAs in the regulation of the epigenetic mechanisms that modulate foetal growth.

## 5. Conclusions

We have found a relationship between tobacco, the hypermethylation of miRNAs (MIR7-1, MIR3918, MIR1244-1, MIR4721, MIR25, MIR93) in foetal cord blood and foetoplacental angiogenic and growth factors in low-birth-weight newborns of smoking mothers.

Further studies are required to shed more light on the effect of tobacco use during pregnancy on silencing due to miRNA methylation and foetoplacental development.

## Figures and Tables

**Table 1:** Main clinical characteristics of the sample.

SEX	Smokig	Gestational Age	Weight	APGAR test	Delivery	Height (cm)	Head Circunference (cm)	Placental Weight (g)
F	Yes	39 + 2	2680	10	Vaginal	46.5	32.5	420
M	Yes	38	2600	10	Cesarean	50	33	530
M	Yes	39 + 1	2725	9	Vaginal	49	33	465
M	Yes	40	2835	10	Vaginal	50	33.5	480
F	Yes	39 + 6	2600	10	Vaginal	50	33	510
F	No	39	2885	10	Vaginal	49	34.5	520
M	No	38	3070	10	Vaginal	51	34	515
F	No	41	4005	9	Vaginal	51	34	635
F	No	41	4100	10	Cesarean	51	35	700
M	No	41	3850	9	Cesarean	51.5	35.5	525

**Table 2:** Hypermethylated miRNA by MedIP-seq.

	miR 7-1 3p	miR 3918	miR 1244-1	miR 4721
Symbols	MIR7-1	MIR3918	MIR1244-1	MIR4721
Alias	hsa-mir-7-1	hsa-mir-3918	hsa-mir-1244-1	hsa-mir-4721
Species	Homo Sapiens	Homo Sapiens	Homo Sapiens	Homo Sapiens
Gene Family	mir-7	mir-3918	mir-1244	mir-4721
Mapped	Chromosome 9 (9q21.32)	Chromosome 6 (6q35.3)	Chromosome 2 (2q37.1)	Chromosome 16 (16P11.2)
Start	83969748	158764661	231713314	28843919
End	83969857 (−)	158764753 (−)	231713398 (+)	28844007 (−)
Gene ID NCBI	407043	100500851	100302285	100616256
Gene Type	ncRNA	ncRNA	ncRNA	ncRNA
Central RNA	URS00002C5007_9606	URS000075B9DC_9606	URS000075BB1F_9606	URS000075EF66_9606
Mature Sequence miRNA	66-CAACAAAUCACAGU CUGCCAUA-87	19-ACAGGGCCGCAGA UGGAGACU-39	55-AAGUAGUUGGUUUGUAUGAGAUGGUU-80	60-UGAGGGCUCCAGGUGACGGUGG-81
TARGET	2037	639	390	390
Promotor miRNA	MI0000263	MI0016424	MI0006379	MI0017356
Mature miRNA	MIMAT0004553	MIMAT0018192	MIMAT0055896	MIMAT0019835
miRDB ID	hsa-mir-7-1-3p	hsa-mir-3918	hsa-mir-1244	hsa-mir-74721
OMIM ID	615239			
HCNG ID	407043	38919	35310	41609
Evidence	Experimental Clonado	Experimental Illumina	Experimental Illumina	Experimental Illumina

**Table 3:** Hypermethylated miRNA by MedIP-seq.

	miR 25 5p	miR 25 3p	miR 93 5p	miR 93 3P	miR 3656
Symbols	MIR25	MIR25	MIR93	MIR93	MIR3656
Alias	hsa-mir-25 5p	hsa-mir-25 3p	hsa-mir-93 5p	hsa-mir-93 3p	hsa-mir-3656
Species	Homo Sapiens	Homo Sapiens	Homo Sapiens	Homo Sapiens	Homo Sapiens
Gene Family	mir-25	mir-25	mir-93	mir-93	mir-3656
Mapped	Chromosome7 (7q22.1)	Chromosome 7 (7q22.1)	Chromosome 7 (7q22.1)	Chromosome 7 (7q22.1)	Chromosome 11 (11q23.3)
Start	100093560	100093560	100093768	100093768	119018944
End	100093643 (−)	100093643 (−)	100093847 (−)	100093847 (−)	119019012 (−)
Gene ID- NCBI	407014	407014	407050	407050	100500840
Gene Type	ncRNA	ncRNA	ncRNA	ncRNA	ncRNA
Central RNA	URS00000C85B2_9606	URS00000C85B2_9606	URS00000DDD35_9606	URS00000DDD35_9606	URS000075C7 FA_9606
Mature Sequence miRNA	14-AGGCGGAGACUU GGGCAAUUG-34	52-CAUUGCACUUGUC UCGGUCUGA-73	11-CAAAGUGCUGUUCG UGCAGGUAG-33	50-ACUGCUGAGCUAGCACUUCCCG-71	49-GGCGGGUGCG GGGGUGG-65
TARGET	51	919	1319	651	
Promotor miRNA	MI0000082	MI0000082	MI0000095	MI0000095	MI0015056
Mature miRNA	MIMAT0004498	MIMAT0000081	MIMAT0000093	MIMAT0004509	MIMAT00180 76
miRDB ID	hsa-mir-25-5p	hsa-mir-25-3p	hsa-mir-93 5p	hsa-mir-93 3p	
OMIM ID	612150	612150	612984	612984	
HCNG ID	407014	407014	407050	407050	38889
Evidence	Experimental Clonado	Experimental Clonado Northern	Experimental Clonado Northern	Experimental Clonado	Experimental

**Table 4:** Targets of miR-7-1.

	Gene Name	GENE ID NCBI
TUSC2	Mitochondrial Calcium Regulator of Tumor Suppressor Gene 2	11334
TNFSF4	Member number 4 superfamily TNF (Tumor Necrosis Factor)	7292
CADM2	cell adhesion molecule 2	253559
VEGFA	Vascular Endothelial Growth Factor A	7422
TP531NP1	Inducible nuclear p53 tumor protein 1 (Tumor Suppressor Gene)	94241
TRAF6	TNF receptor (Tumor Necrosis Factor) associated with factor 6	7189
BCCIP	BRCA2 interaction protein (Tumor Suppressor Gene)	56647
TRAF7	Factor 7 associated with the TNF Receptor (Tumor Necrosis Factor)	84231
AMMECR1	Alport syndrome. Gen 1	9949
CDC23	Cell Division Cycle Protein 23	8697
CDC73	Protein 73 Cell Division Cycle	79577
BCL2L1	BCL2-like 1 (Apoptosis Inhibitory Protein)	598
MORF4L2	Mortality Factor 4-like 2	
CDC14A	Cell Division Cycle 14A	8556
CDC37L1	Cell Division Cycle 37 like 1	55664
CLLU1OS	Chronic Lymphocytic Leukemia, positive regulation of the opposite chain	574016
FMR1	Fragile X syndrome	2332
RAP1B	Member of the RAS oncogene family	9643

**Table 5:** Targets of miR-3918.

	Gene Name	GENE ID NCBI
RAB5B	RAB5B Member of the RAS oncogene family	5869
BAP1	Protein associated with BRCA1 (Tumor Suppressor Gene)	8314
TRARG1	GLUT4 Receptor Regulator (Glucose transporter protein regulated by insulin)	286753
PGF	Placental Growth Factor	5228
CDON	Cell Adhesion Regulatory Oncogene	50937
RAB4B	RAB4B Member of the RAS oncogene family	53916
CSHL1	Human Chorionic Somatomammotropin Hormone-like 1	1444
TNFRSF18	Member 18 TNF (Tumor Necrosis Factor) receptor superfamily	8784
OSGIN1	Inhibitor 1 of the Oxidative Stress Induction Factor	29948
TNFAIP2	TNF α-inducing protein 2 (Tumor Necrosis Factor alpha)	7127
TNFAIP1	TNF α-inducing protein 1 (Tumor Necrosis Factor alpha)	7126
SOHLH1	Specific Basic Protein of spermatogenesis and oogenesis	402381
NBAS	Neuroblastoma amplified sequence	51594
RAB40C	RAB40C member of the RAS oncogene family	57799
IGF2BP2	IGF2 mRNA-binding protein 2 (Insulin Growth Factor 2)	10644

**Table 6:** Targets of miR-1244-1.

Target	Gene Name	GENE ID NCBI
F8	Coagulation Factor VIII	2157
NCR3LG1	Natural Killer Cell Receptor 3 Ligand 1	374383
ATXN1L	Ataxin-like 1	342371
MYBL1	MYB-like 1 proto-oncogene	4603
TGFBR2	TGFB Receptor 2 (Transforming Growth Factor beta)	7048
TNFAIP8L2	TNF α (Tumor Necrosis Factor alpha) induced protein 8 like 2 that	79626
DNMT3A	DNA Methyltransferase 3α (alpha)	1788
NEUROD4	Neuronal differentiation 4	58158
HGF	Liver Growth Factor	3082
OSGIN2	Member 2 of the stress-induced growth factor inhibitor family	734
CIP2A	oxidative	57650
FGFR1OP2	Inhibitor of protein phosphatase 2A that regulates cell proliferation	26127
TDGF1	FGFR1 oncogene partner 2	6997
CDC34	Growth Factor 1 derived from teratocarcinoma	997
F3	Cell division cycle factor 34	2152
FXR1	Tissue coagulation factor III	8087
ANAPC11	Autosomal homologous protein 1 of FMR1	51529
CBL	Subunit 11 of the anaphase promoting complex	867

**Table 7:** Targets of miR-4721.

	Gene Name	GENE ID NCBI
TP63	Tumor protein p63	8626
MLLT11	MLLT11 transcription factor cofactor 7	10962
RRAS2	Related to RAS 2	22800
FXR1	Autosomal homologous protein 1 of FMR1	8087
BCAM	Basal cell adhesion molecule	4059
CDCA7	Factor 7 associated with the cell division cycle	83879
MYCL	MYCL proto-oncogene transcription factor	4610
AREL1	Apoptosis resistant protein E3 ubiquitin ligase 1	9870
HIF3A	Hypoxia-inducible factor 3 alpha subunit	64344
ATXN7L3	Ataxin 7 like 3	56970
FXR2	Autosomal homologous protein 2 of FMR1	9513
C1QTNF6	CIQ factor related to TNF (Tumor Necrosis Factor) 6	114904
TNFAIP1	TNF α-inducing protein 1 (Tumor Necrosis Factor alpha)	7126
BAP1	BRCA1 proto-oncogene-associated protein 1	8314

**Table 8:** Targets of miR-25-5p.

Target	Gene Name	GENE ID NCBI
TP63	p63 tumor protein	8626
MISP3	MISP (Mitotic Spindle Positioning) family member 3	133230
APBB2	Binding protein, member 2, family B, of amyloid beta precursor	323
PAN01	Nucleolar proapoptotic protein 1	101927423

**Table 9:** Targets of miR-25-3p.

	Gene Name	GENE ID NCBI
RAB23	RAB23 member of the RAS oncogene family	51715
FBN1	Fibrillin 1	2200
BTG2	BTG antiproliferative protein factor 2	7832
CADM2	Cell adhesion molecule 2	253559
BCL2L11	BCL2-like 11 protein	10018
ATXN1	Ataxin 1	6310
ATXN3	Ataxin 3	4287
ATXN7	Ataxin 7	6314
APBB2	Binding protein, member 2, family B, of amyloid beta precursor.	323
RAB3C	RAB3C RAS oncogene family member	115827
NF2	Neurofibromin 2	4771
WASL	Wiskott-Aldrich syndrome	8976
FXR1	Autosomal homologous protein 1 of FMR1	8087
INSIG1	Insulin-inducing gene 1	3628
RAB8B	RAB8B RAS oncogene family member	51762
IRS2	Insulin receptor substrate 2	8660
FMR1	Fragile X syndrome	2332
SCAI	Cellular invasion suppressor in cancer	286205
CADC42	Cell division cycle factor 42	998
TRAF3	Factor 3 associated with TNF (Tumor Necrosis Factor)	7187
AGGF1	Angiogenic Factor 1	55109
RAB2C	RAP2C member of the RAS oncogene family	57826
VEZF1	Vascular Endothelium-Associated Zinc Finger Protein	7716

**Table 10:** Targets of miR 93-5p.

Target	Gene Name	GENE ID NCBI
ITPRIPL2	ITPRIP-like 2 protein	162073
ATXN1	Ataxin 1	6310
TNFRSF21	Receptor member 21 of the TNF superfamily (Tumor Necrosis Factor)	27242
F3	Coagulation tissue factor III	2152
RAB5B	RAB5B Member of the RAS oncogene family	5869
LRP8	LDL receptor protein 8 (Low Density Lipoprotein)	7804
APP	Beta Amyloid Precursor Protein	351
TPRG1L	Tumor protein p53-like 1 regulator	127262
LRPAP1	Protein 1 associated with the LDL receptor (Low Density Lipoprotein)	4043
CDC23	Cell Division Cycle Protein 23	8697
VASP	Vasodilation Stimulating Phosphoprotein	7408
IGF2BP1	Insulin-like growth factor 2 mRNA-binding protein 1	10642
VEGFA	Vascular Endothelial Growth Factor A (VEGF)	7422
VEZF1	Vascular Endothelium-Associated Zinc Finger Protein	7716
FXN	Frataxin protein	2395
VLDLR	VLDL receptor (Very Low Density Lipoprotein)	7436

**Table 11:** Targets of miR 93-3p.

Target	Gene Name	GENE ID NCBI
RAB36	RAB36 Member of the RAS oncogene family	9609
RAB3B	RAB3B Member of the RAS oncogene family	5865
HYPK	Huntingtin-associated protein K	25764
LDLRAD4	Domain 4 of the VLDL (Very Low Density Lipoprotein) class A receptor	753
RAB4A	RAB4A Member of the RAS oncogene family	5867
PLAC8	Placenta Specific Protein 8	51316
CDC14B	Cell cycle-associated factor 14B	8555
HIVEP3	HIV (Human Immunodeficiency Virus) type I binding protein 3	59269
TRAF1	Factor 1 associated with TNF (Tumor Necrosis Factor)	7185

## Data Availability

The data presented in this study are available on request from the corresponding author.
